# Research hotspots and future trends in sepsis-associated acute kidney injury: a bibliometric and visualization analysis

**DOI:** 10.3389/fmed.2024.1456535

**Published:** 2025-01-07

**Authors:** Xing-Yue Chen, Li-Jia Zhi, Jun Chen, Rong Li, Kun-Lan Long

**Affiliations:** ^1^Department of Critical Care Medicine, Hospital of Chengdu University of Traditional Chinese Medicine, Chengdu, China; ^2^School of Clinical Medicine, Chengdu University of Traditional Chinese Medicine, Chengdu, China

**Keywords:** bibliometrics, sepsis, acute kidney injury, CiteSpace, VOSviewer

## Abstract

**Objectives:**

Sepsis-associated acute kidney injury (SA-AKI) commonly occurs in critically ill patients and is closely associated with adverse outcomes. A comprehensive analysis of the current research landscape in SA-AKI can help uncover trends and key issues in this field. This study aims to provide a scientific basis for research directions and critical issues through bibliometric analysis.

**Methods:**

We searched all articles on SA-AKI indexed in the SCI-Expanded of WoSCC up to May 7, 2024, and conducted bibliometric and visual analyses using bibliometric software CiteSpace and VOSviewer.

**Results:**

Over the past 20 years, there has been a steady increase in literature related to renal repair following AKI. China and the United States contribute over 60% of the publications, driving research in this field. The University of Pittsburgh is the most active academic institution, producing the highest number of publications. J. A. Kellum is both the most prolific and the most cited author in this area. “Shock” and “American Journal of Physiology-Renal Physiology” are the most popular journals, publishing the highest number of articles. Recent high-frequency keywords in this field include “septic AKI,” “mitochondrial dysfunction,” “inflammasome,” “ferroptosis,” and “macrophage.” The terms “mitochondrial dysfunction,” “inflammasome,” “ferroptosis,” and “macrophage” represent current research hotspots and potential targets in this area.

**Conclusion:**

This is the first comprehensive bibliometric study to summarize the trends and advancements in SA-AKI research in recent years. These findings identify current research frontiers and hot topics, providing valuable insights for scholars studying SA-AKI.

## 1 Introduction

Sepsis is defined by a dysregulated host response to infection, resulting in life-threatening organ dysfunction, frequently encompassing acute kidney injury (AKI) (1). Sepsis represents 45–70% of all AKI cases among critically ill patients (2, 3). Sepsis-associated acute kidney injury (SA-AKI) is prevalent in this population and is closely linked to detrimental outcomes, including an elevated risk of chronic kidney disease, cardiovascular events, and mortality. The current optimal definition of SA-AKI is the onset of AKI within 7 days following the onset of sepsis, diagnosed in accordance with the Kidney Disease Improving Global Outcomes (KDIGO) criteria (4) and the Third International Consensus Definitions for Sepsis and Septic Shock (Sepsis-3) criteria (5). Despite decades of investigation, the pathophysiology of sepsis-induced AKI remains inadequately elucidated. Historically, sepsis-induced AKI was regarded as a renal circulatory disease (6), attributed to global renal ischemia, cellular injury, and acute tubular necrosis (ATN). Increasing evidence indicates that AKI can manifest in a subset of patients without overt signs of perfusion deficit, suggesting the involvement of alternative mechanisms (7). Numerous facets of SA-AKI remain poorly characterized, including its epidemiology, pathophysiology, the impact of resuscitation and fluid strategies, the role of biomarkers in risk stratification and diagnostic and therapeutic guidance, as well as the effects of extracorporeal therapies and novel treatments on patient outcomes. Hence, a comprehensive understanding of the current research landscape and emerging trends concerning SA-AKI is imperative. Bibliometric analysis entails the quantitative examination of bibliographic materials through mathematical and statistical methods, facilitating the analysis of developmental and research patterns in specific fields (8). It grants researchers a broad perspective on essential data and dynamic trends, aiding in the evaluation of the quantity and quality of existing issues, institutions, and regional publications (9). Furthermore, bibliometrics is indispensable in forecasting potential future research directions and developmental trajectories. Thus, it is widely utilized and regarded as an essential tool for research assessment. Despite the substantial volume of literature on SA-AKI published to date, no study has yet performed visual analyses employing bibliometric methods.

## 2 Materials and methods

### 2.1 Data source and search strategy

**Figure 1:**
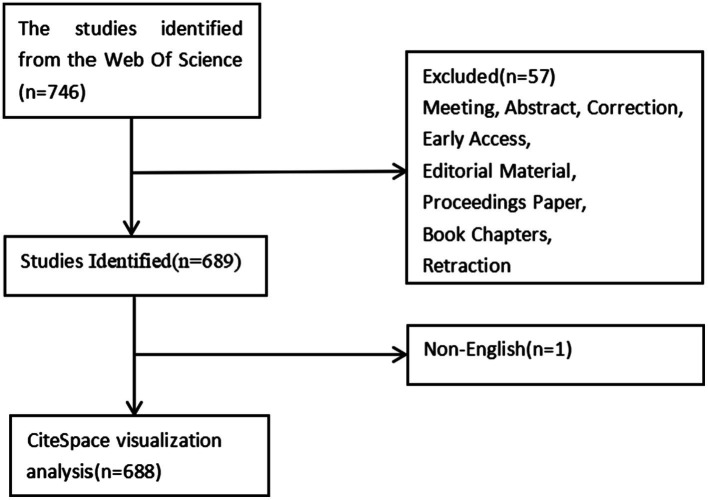
Flowchart of the screening process.

This study employed the Science Citation Index Expanded from the Web of Science Core Collection (Clarivate Analytics) as the literature retrieval database, renowned for its systematic, authoritative, and comprehensive nature, making it a preferred choice for bibliometric and visualization analyses (10, 11). Given the swift updates to the database’s content, all data were independently retrieved by two authors within a single day, specifically May 7, 2024, to ensure real-time accuracy. The search keywords employed were “TS = (sepsis-induced acute kidney injury) OR TS = (sepsis-associated acute kidney injury) OR TS = (septic-induced acute kidney injury) OR TS = (septic-associated acute kidney injury) AND Document types = (ARTICLE OR REVIEW) AND Language = (English).” The retrieval period spanned from January 1, 2002, to May 7, 2024. Furthermore, all pertinent bibliographic data—including publication year, title, authors’ names, nationality, affiliations, abstracts, keywords, and journal titles—were meticulously stored in plain text format within the WoSCC database. Titles and abstracts of retrieved publications were also manually reviewed and screened to further exclude retractions and irrelevant records. The inclusion and exclusion criteria for the literature are depicted in Figure 1.

### 2.2 Data analysis

The literature downloaded for this study will subsequently be subjected to an analysis employing widely utilized bibliometric analysis tools, including CiteSpace 6.1.3, VOSviewer 1.6.18 (Leiden University Center for Science and Technology Studies), and R’s Biblioshiny platform. VOSviewer 1.6.18 is a sophisticated bibliometric analysis software adept at extracting critical information from an array of publications (12), frequently utilized for constructing collaboration, co-citation, and co-occurrence networks (13, 14). In our inquiry, the software will primarily facilitate the following analyses: examination of countries and institutions, analysis of journals and co-cited journals, assessment of authors and co-cited authors, as well as keyword co-occurrence analysis. In the visualizations generated by VOSviewer, each node symbolizes an entity, be it a country, institution, journal, or author. The size and hue of nodes, respectively, denote the quantity and category of these entities. The thickness of the lines interconnecting nodes reflects the intensity of collaboration or co-citation (15, 16). CiteSpace 6.1.3, developed by Professor Chen C., is yet another software instrument employed for bibliometric analysis and visualization (17, 18). In this study, CiteSpace will be utilized to generate overlay dual-map journal visualizations and to conduct burst detection analyses on keywords (19), wherein burst strength indicates the frequency of keyword occurrences (20). Start and end times delineate the temporal distribution of keywords, while hotspots are characterized as high-frequency keywords within prominent scientific domains (21). The R package “bibliometrix” (version 3.2.1)^1^ will be employed to scrutinize collaboration between countries and regions. Furthermore, Microsoft Office Excel 2019 will be harnessed for the quantitative analysis of publications.

## 3 Results

### 3.1 Annual publication outputs

**Figure 2:**
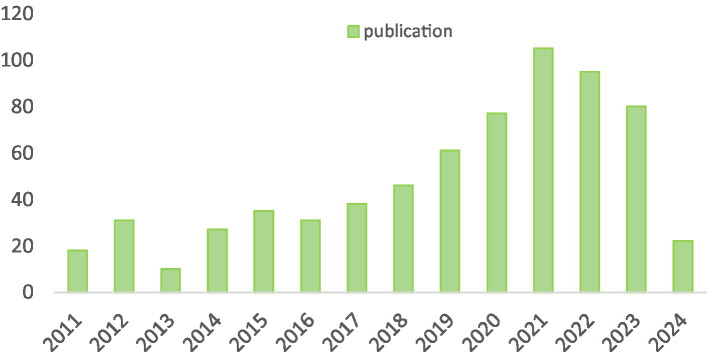
Annual research output on SA-AKI.

In accordance with our investigative strategy, over the past two decades, a total of 615 articles and 73 reviews concerning SA-AKI have been published. We scrutinized the annual publication figures, with the earliest pertinent article traced back to 2011. As illustrated in Figure 2, the entirety of this time frame can be segmented into three distinct phases: Phase 1 (2011–2016), Phase 2 (2017–2021), and Phase 3 (2022–2023). During Phase 1, research was in its nascent stages, averaging 25.3 articles per year. Commencing in 2017, there was a marked surge, culminating in 105 articles by 2021. Nevertheless, in the subsequent 2 years, a slight regression has been observed, with an average of approximately 87.5 articles per year, although the subject of SA-AKI continues to be a prominent focus of inquiry.

### 3.2 Country and institutional analysis

These publications emanate from 45 nations and 200 institutions. The foremost 10 countries (as detailed in Table 1) exhibiting the highest number of publications are spearheaded by China (*n* = 397, 49.7%), followed by the United States (*n* = 120, 15.0%), India (*n* = 21, 2.6%), and Germany (*n* = 21, 2.6%). China constitutes nearly half of the total publications, with the United States following as the second most significant contributor.

**Table 1:** Top 10 countries and institutions in SA-AKI research.

Country	Count	Institution	Count
China	397	University of Pittsburgh	22
The United States	120	Wuhan University	21
Italy	21	Southern Medical University	21
Germany	21	Shanghai Jiaotong University	21
Brazil	18	Nanjing Medical University	19
Japan	17	Capital Medical University	17
Egypt	16	Huazhong University of Science and Technology	14
Korea	15	University of Melbourne	12
Australia	15	Central South University	12
Thailand	12	Wenzhou Medical University	11

**Figure 3:**
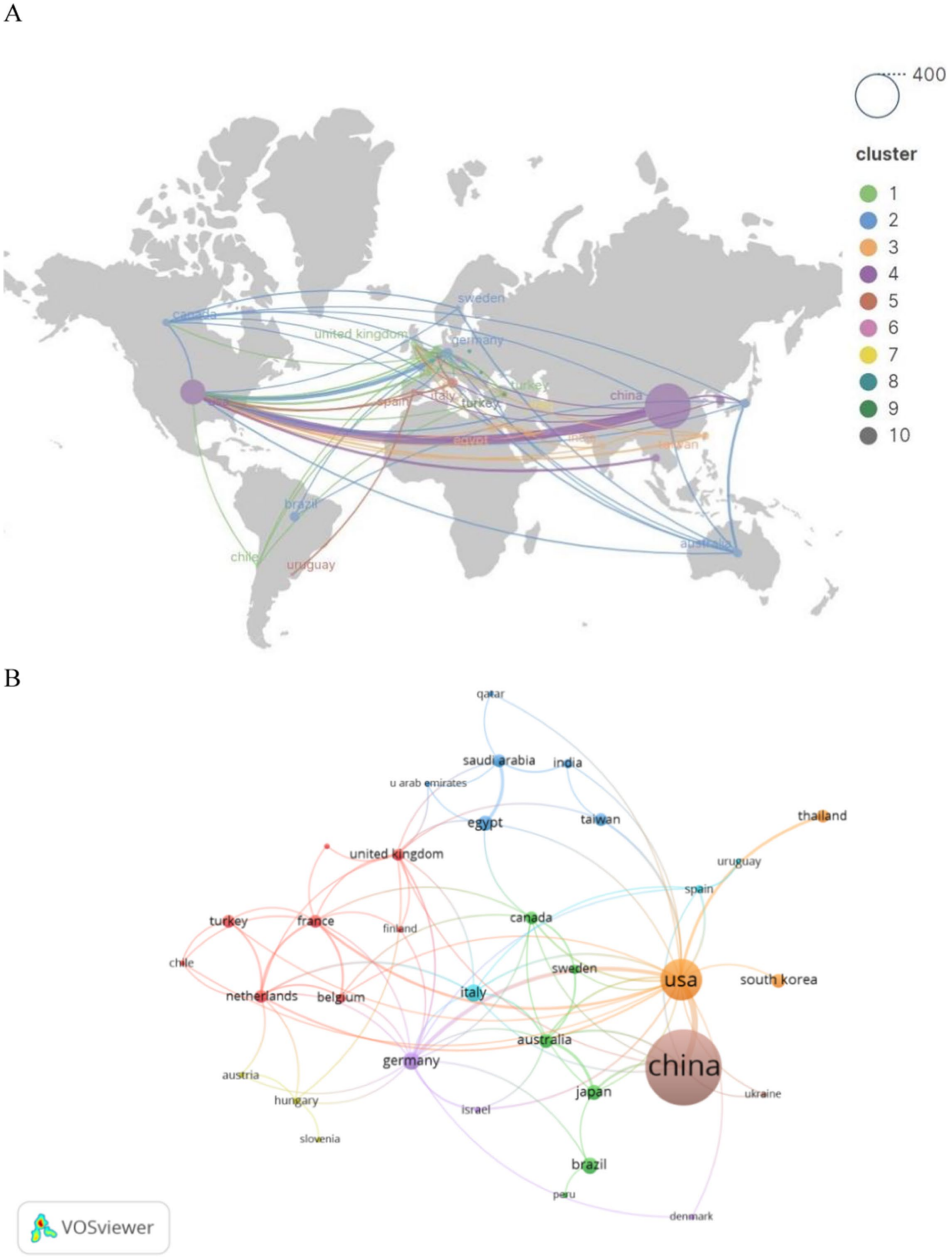
Geographic distribution **(A)** and country visualization **(B)** of SA-AKI research.

Subsequently, we filtered and visualized the 45 nations based on publications of two or more, and constructed a collaborative network (Figure 3) reflecting the quantity of publications and interrelations among each country. It is particularly noteworthy that there exists a plethora of active collaborations among various nations. For instance, China maintains a close collaborative relationship with the United States, Japan, and Australia; the United States actively engages with Germany, Thailand, and France.

**Figure 4:**
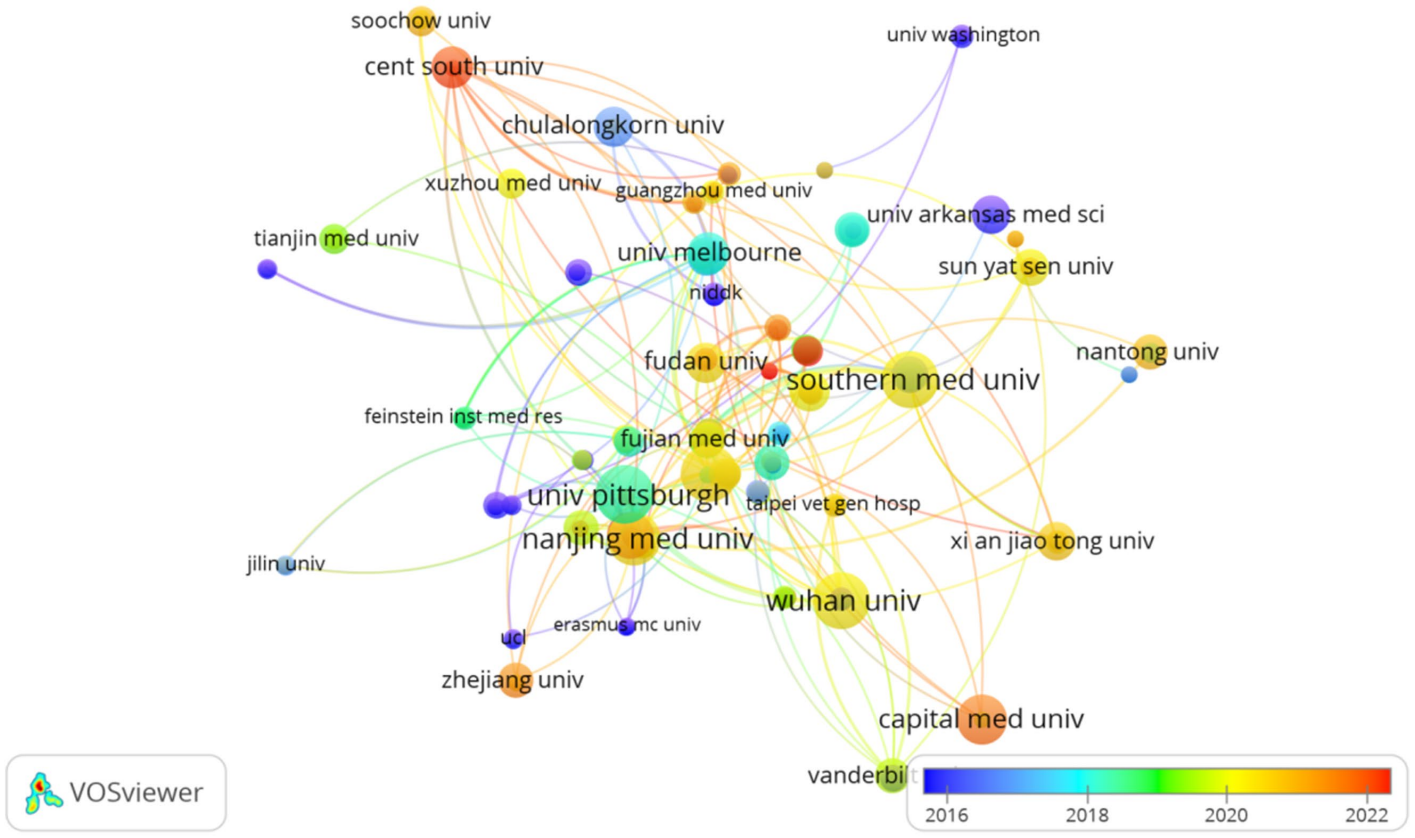
Institutional visualization of SA-AKI research.

The top 10 universities hail from three countries, with four out of five situated in China. The five universities that have published the most pertinent papers are: University of Pittsburgh (*n* = 22, 2.8%), Wuhan University (*n* = 21, 2.6%), Southern Medical University (*n* = 21, 2.6%), Shanghai Jiao Tong University (*n* = 21, 2.6%), and Nanjing Medical University (*n* = 19, 2.4%). Subsequently, we selected 200 institutions for visualization based on the criterion of having published at least 2 papers, constructing a collaborative network (Figure 4) based on the volume of publications and interrelationships for each institution. From Figure 4, it is evident that Wuhan University collaborates closely with Nanjing Medical University and Southern Medical University, while the Feinstein Institutes for Medical Research forges strong partnerships with the University of Melbourne and the University of Pittsburgh.

### 3.3 Journals and co-cited journals

**Figure 5:**
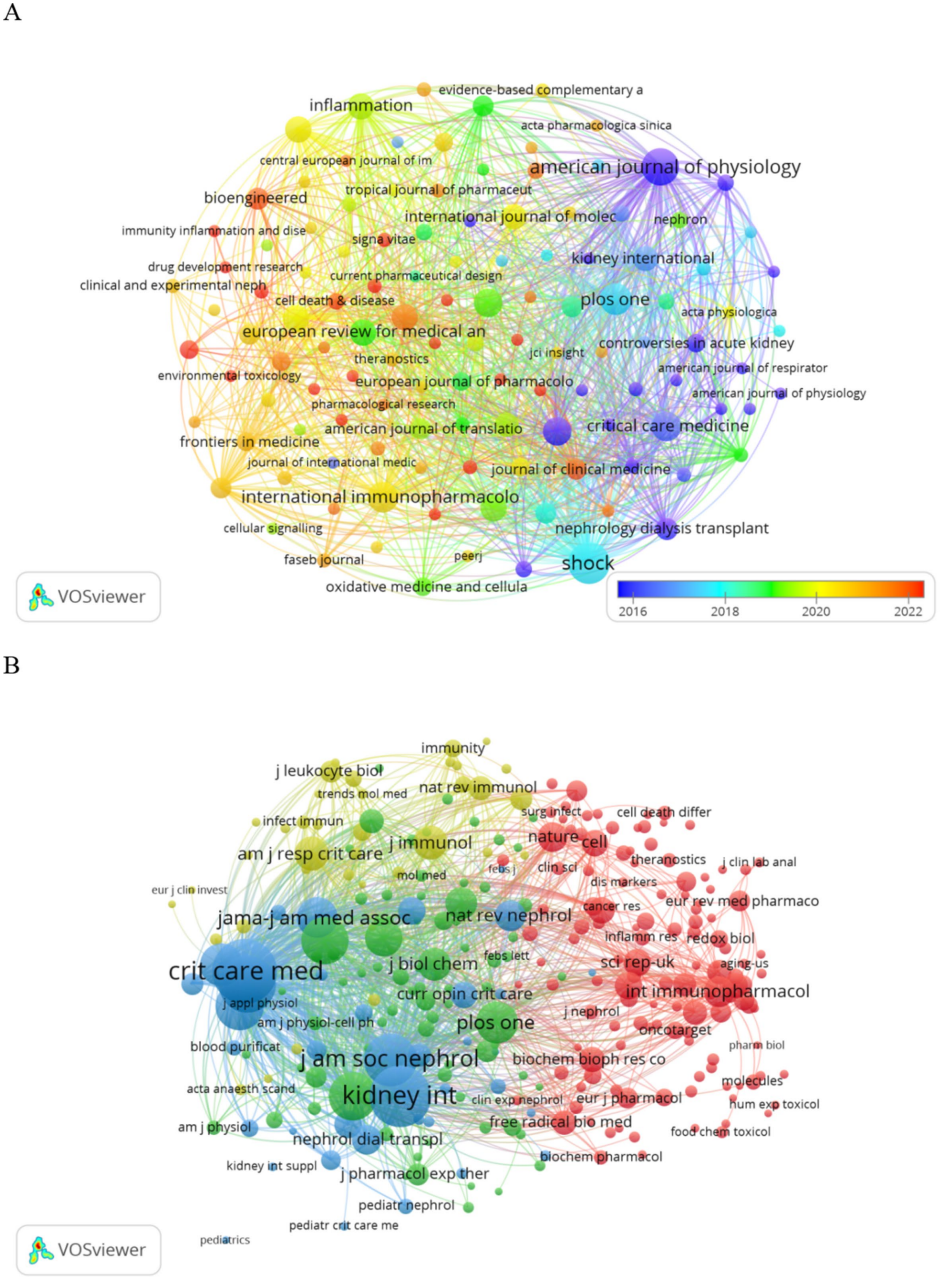
Visualization of SA-AKI research journals **(A)** and co-cited journals **(B)**.

Publications pertaining to SA-AKI have been disseminated across 200 journals. As indicated in Table 2, the journal with the highest number of publications is Shock (*n* = 24, 3.6%), followed by the American Journal of Physiology-Renal Physiology (*n* = 19, 2.8%), PLoS One (*n* = 14, 2.1%), and International Immunopharmacology (*n* = 13, 1.9%). Among the top 15 journals ranked by impact factor, “Kidney International” holds the highest impact factor (IF = 19.6), trailed by “Critical Care” (IF = 15.1). Subsequently, we selected journals based on the criterion of a minimum of two related publications and constructed a journal citation network graph (Figure 5A). Figure 5A illustrates the close citation relationships between the American Journal of Physiology-Renal Physiology and journals such as Critical Care Medicine, Kidney International, and PLoS One.

**Table 2:** Contributions of the top 10 journals on SA-AKI.

Rank	Journal	Publications	Citations	The percentage of articles of institutions in total publications	IF
1	Shock	24	1,106	3.6	3.1
2	American Journal of Physiology-Renal Physiology	19	702	2.8	4.2
3	PLoS One	14	459	2.1	3.7
4	International Immunopharmacology	13	420	1.9	5.6
5	Critical Care Medicine	12	404	1.9	8.8
6	Critical Care	11	732	1.6	15.1
7	Biomedicine & Pharmacotherapy	11	281	1.6	7.5
8	Life Sciences	10	371	1.5	6.1
9	Renal Failure	10	206	1.5	3.0
10	Frontiers in Immunology	10	203	1.5	7.3

Among the top 15 cited journals, six have been referenced more than 500 times each. The most cited journal is Shock (cited 1,106 times), followed by Kidney International (cited 1,074 times), Critical Care (cited 732 times), and the American Journal of Physiology-Renal Physiology (cited 702 times). Journals were selected based on a minimum of 20 citations, and a citation network graph was constructed (Figure 5B). As depicted in Figure 5B, Critical Care Medicine exhibits positive citation relationships with journals such as Nature Reviews Nephrology and Kidney International.

**Figure 6:**
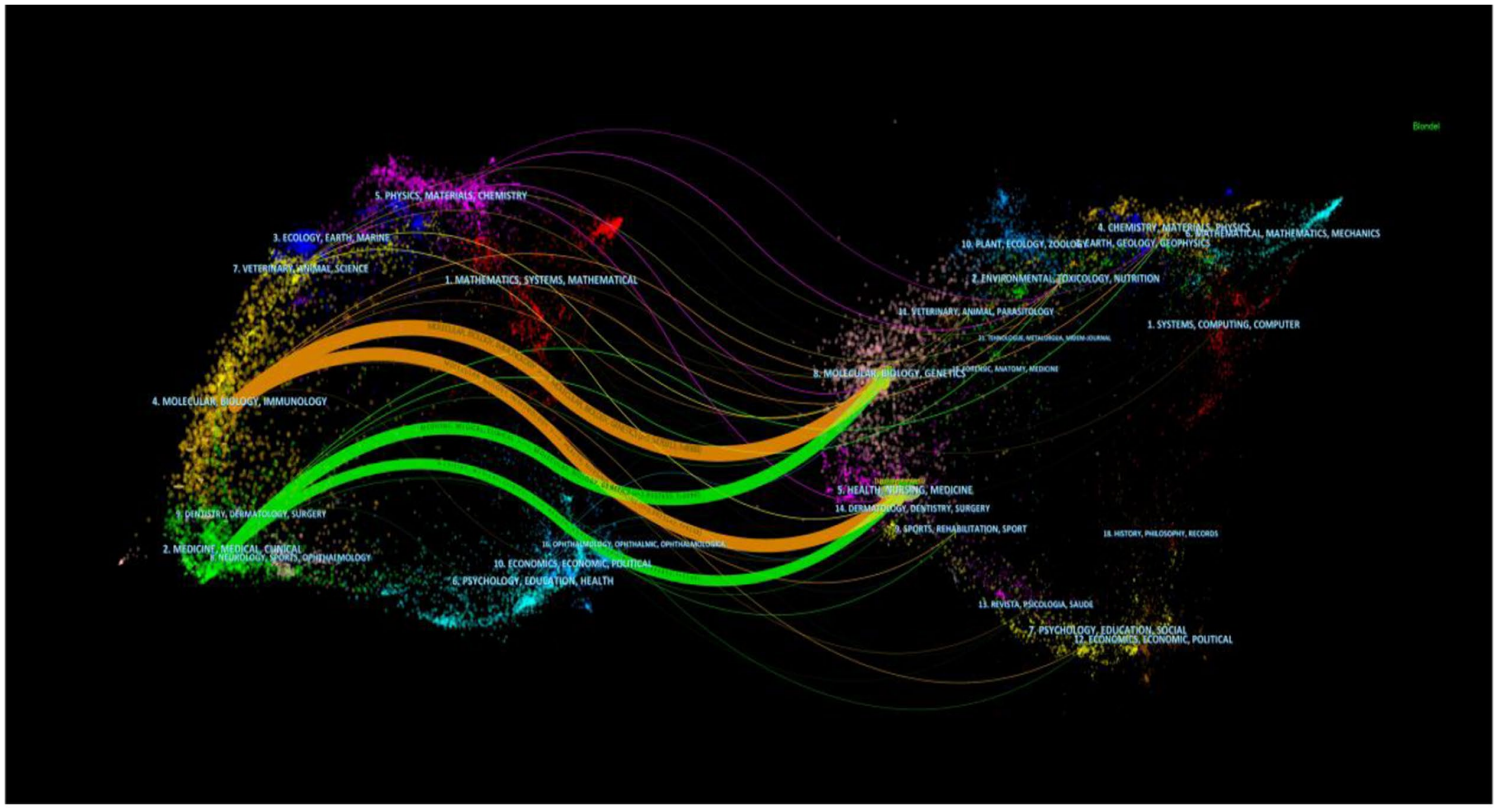
Overlay of SA-AKI research journals.

The dual overlay display of the journal elucidates the distribution of its themes (Figure 6). Cited journals are positioned on the left side of the map, while citing journals are situated on the right. The labels signify the disciplines encompassed by the journals. From left to right, the colored lines portray the citation pathways, delineating four distinct citation trajectories (22). The orange pathway represents the primary citation route, indicating that the research published in the Molecular/Biology/Genetics journal is frequently referenced in studies conducted within the Molecular/Biology/Immunology journal.

### 3.4 Authors and co-cited authors

**Figure 7:**
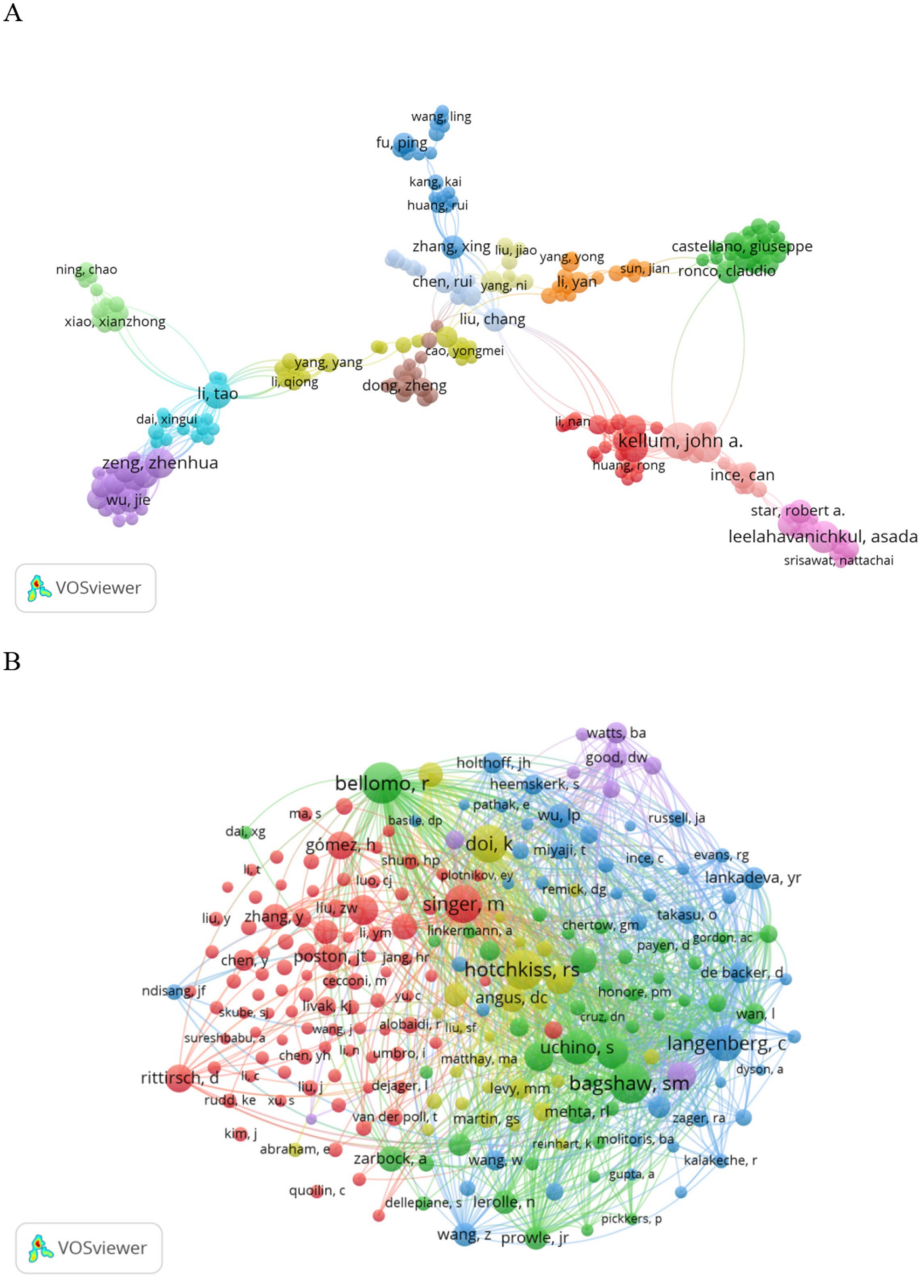
Visualization of authors **(A)** and co-cited authors **(B)** in SA-AKI research.

In 1919, authors contributed to SA-AKI research. Twelve authors published five or more papers (see Table 3). A collaboration network was established among authors with two or more publications (refer to Figure 7A), wherein the nodes for Kellum J. A., Bellomo R., Leelahavanichkul A., and Clive N. May stand out due to their prolific output. Furthermore, close collaborations were observed among several authors; for instance, Xing Zhang maintained a close association with Rui Huang, Rui Chen, Yang Ni, and others.

**Table 3:** Top 10 authors by publication count in SA-AKI research papers.

Rank	Author	Publications	Citations
1	Kellum J. A.	14	1,969
2	Bellomo R.	12	399
3	Leelahavanichkul A.	11	472
4	Clive N. May	11	322
5	Mayeux P. R.	10	693
6	Zeng Zhenhua	11	732
7	Hernando Gomez	9	1,758
8	Li Tao	9	212
9	Peng Zhiyong	9	145
10	Chen Zhongqing	8	163

Additionally, we noted the total citations of authors, reflecting their recognition within the field. Kellum J. A., with 1,969 citations, garnered the highest total citations. Gomez H. ranked second in total citations. Authors with a minimum co-citation count of 15 were selected to construct a co-citation network (see Figure 7B). As illustrated in Figure 7B, active collaborations exist among various co-cited authors; for example, Bellomo R. closely collaborated with Bagshaw S. M., Uchino S., and others.

### 3.5 Co-cited references

**Figure 8:**
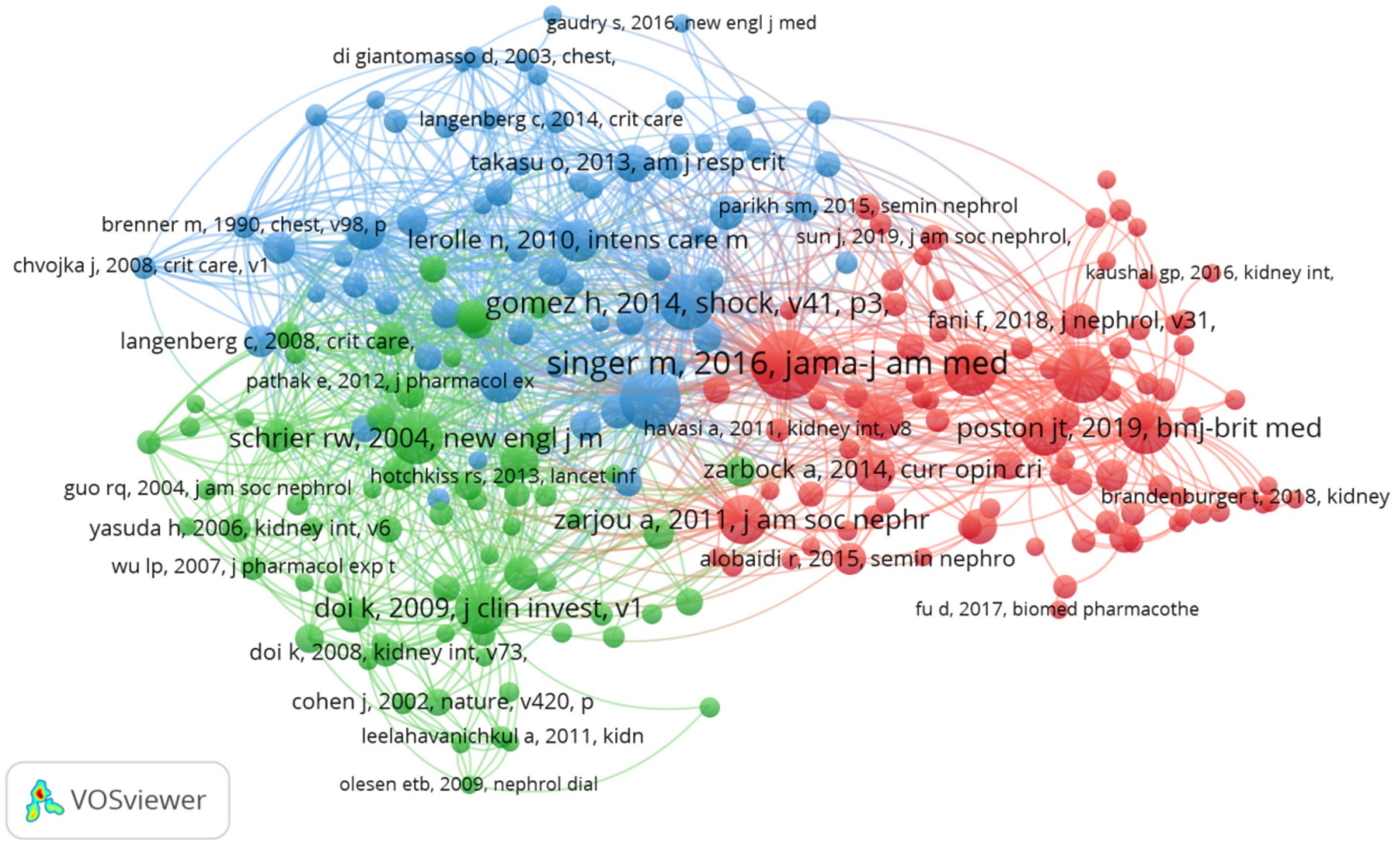
Visualization of co-cited publications in SA-AKI research.

Over the past two decades, research concerning SA-AKI has been referenced in a cumulative total of 22,052 scholarly publications. We devised a co-citation network (Figure 8) utilizing publications that have been cited 10 or more times. From Figure 8, it becomes apparent that there exist strong co-citation relationships among works such as “Singer M., 2016, JAMA” in conjunction with “Poston J. T., 2019, BMJ,” “Zarjou A., 2011, J Am Soc Nephrol,” “Gomez H., 2014, Shock,” among others.

### 3.6 Reference with citation bursts

**Figure 9:**
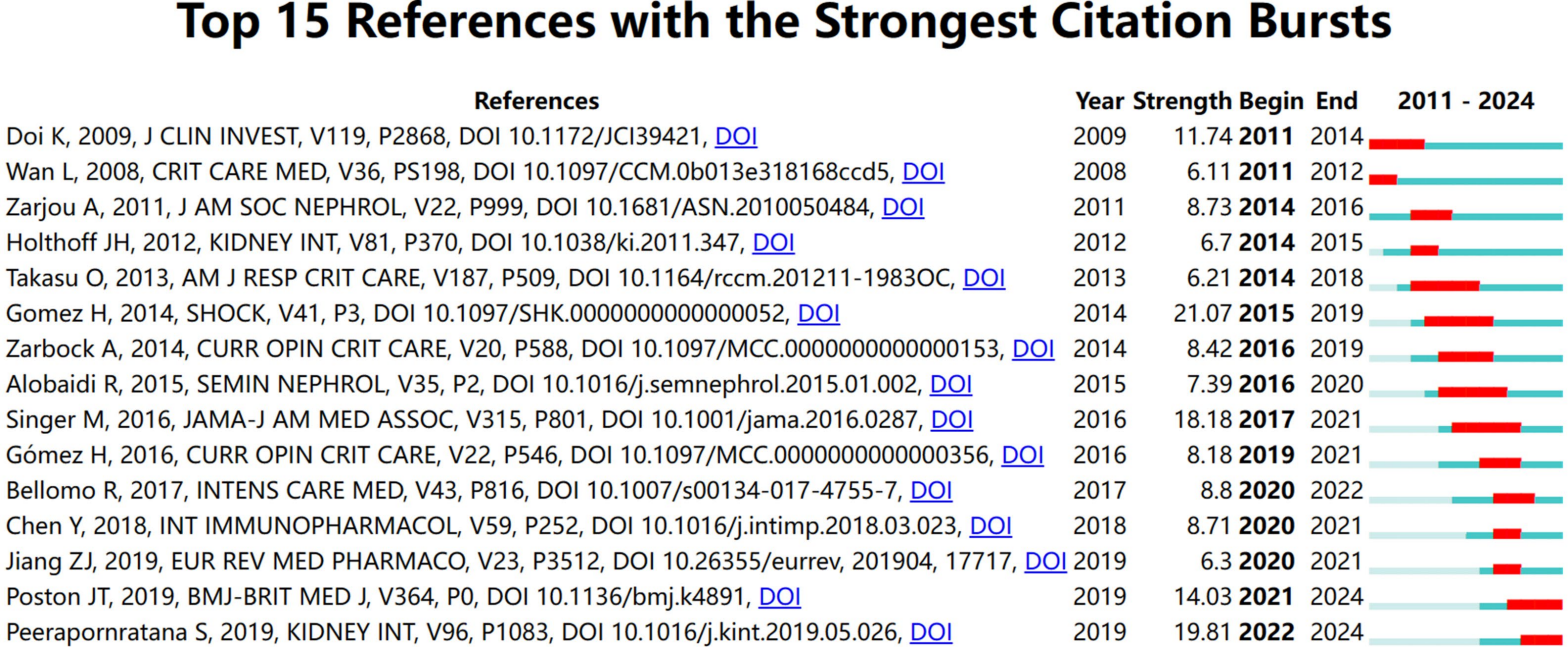
Top 15 references with strong citation bursts.

We utilized CiteSpace to select highly cited references. The results reveal that over time, the following 15 references have been extensively cited (Figure 9), with their findings being well recognized in the field. The deep blue line denotes the citation duration from 2011 to 2024, while the red bars illustrate the intensity of citation bursts (23). Citation bursts in the references were observed as early as 2008 and as recently as 2024. The most robust citation burst (intensity = 21.07) was noted in the article titled “A unified theory of sepsis-induced acute kidney injury: inflammation, microcirculatory dysfunction, bioenergetics, and the tubular cell adaptation to injury,” authored by Gomez H. et al., with citation bursts occurring from 2014 to 2019. The second highest citation burst (intensity = 19.81) was identified in the article titled “Acute kidney injury from sepsis: current concepts, epidemiology, pathophysiology, prevention, and treatment” by Sadudee Peerapornratana et al., published in Kidney International, with citation bursts occurring from 2011 to 2019. Overall, citation burst intensities for the top 15 articles ranged from 6.11 to 21.07.

### 3.7 Hotspots and frontiers

**Figure 10:**
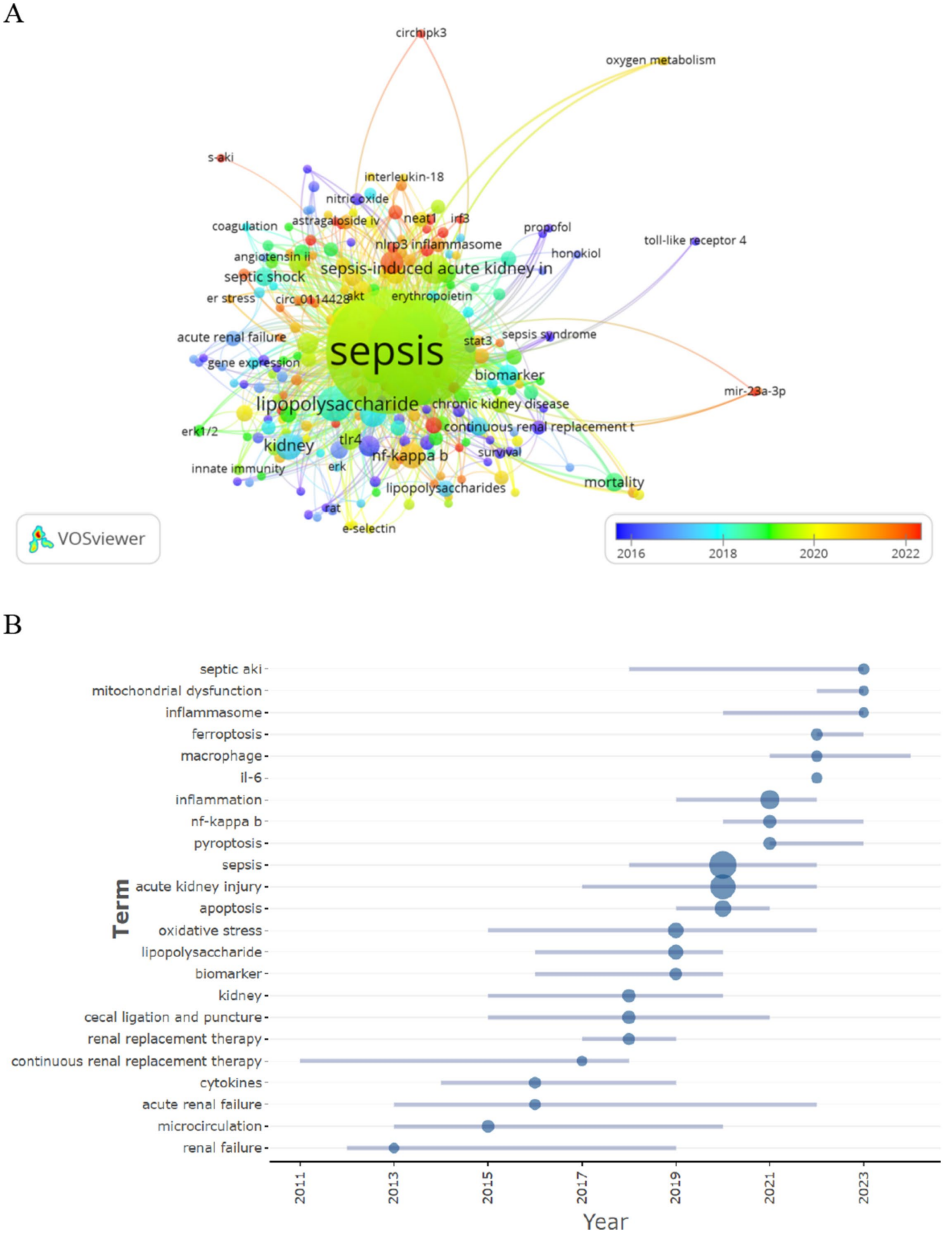
Keyword clustering analysis **(A)** and trend topic analysis **(B)**.

Through the analysis of co-occurring keywords, one can swiftly discern research hotspots within a particular domain. We filtered keywords that appeared two or more times and performed a cluster analysis utilizing VOSviewer (see Figure 10A). The thickness of the lines connecting nodes denotes the strength of relationships between the keywords. Ultimately, we identified three distinct clusters delineating three research directions.

The green cluster comprises keywords such as sepsis, sepsis-induced acute kidney injury, innate immunity, and Extracellular Signal-regulated Kinase 1/2. The red cluster encompasses terms like S-AKI, nlrp3, and mir-22-3p, among others. Keywords in the blue cluster include renal replacement therapy, nitric oxide, toll-like receptor 4, and various others. The keyword trend topic analysis (refer to Figure 10B) elucidates the temporal trajectory and evolution of specific research themes. Presently, the investigation into SA-AKI predominantly revolves around mitochondrial dysfunction, inflammasome activity, ferroptosis, and macrophage involvement.

## 4 Discussion

### 4.1 General information

Beginning in 2011, scholarly articles on SA-AKI began to emerge gradually. The publication of the KDIGO guidelines (24) offered a foundational basis for research on SA-AKI; however, until 2016, this area of inquiry remained in its nascent stages, with an average of merely 25.3 articles published annually. From 2017 to 2021, the volume of published works experienced a significant upsurge, averaging 65.4 articles per year. This remarkable increase in publication numbers may correlate with the dissemination of various guidelines (1, 25–28). These guidelines and expert consensus have provided crucial guidance for clinical trials and established diagnostic criteria for SA-AKI. Despite a noticeable decline in publication volume over the past 2 years, the output remains substantial, averaging approximately 87.5 articles annually. Currently, research on SA-AKI remains a vibrant topic, garnering increasing attention from scholars.

An analysis of national and institutional distribution aids in fostering collaboration within research teams both domestically and globally. China emerges as a formidable leader in SA-AKI research, boasting the highest publication output, followed by the United States (*n* = 120, 15.0%) and India (*n* = 21, 2.6%). China maintains close partnerships with the United States, Japan, and Australia; similarly, the United States actively engages in collaborations with Germany, Thailand, and France. Among the top 10 research institutions, approximately 80% are situated in China, with the University of Pittsburgh (*n* = 22, 2.8%) contributing the most extensive body of SA-AKI-related research. Numerous institutions demonstrate strong collaborative ties; for example, Wuhan University collaborates intimately with Nanjing Medical University and Southern Medical University, while the Feinstein Institutes for Medical Research maintain a close partnership with the University of Melbourne and the University of Pittsburgh.

While certain nations display commendable collaborative endeavors, the scope and profundity of institutional cooperation remain regrettably inadequate. Undoubtedly, the augmentation of enduring institutional collaboration is advantageous for the progression of research within this realm. Therefore, we fervently implore research institutions globally to partake in expansive collaboration and exchange, thereby collectively nurturing the advancement of SA-AKI. The journal with the most publications concerning SA-AKI research is “SHOCK” (IF = 3.1, Q2), which currently stands as the most esteemed journal in this academic field. The journal boasting the highest impact factor is “Kidney International” (IF = 19.6, Q1), succeeded by “Critical Care” (IF = 15.1, Q1). An examination of co-cited journals reveals that the majority are esteemed Q1 publications, clearly indicating their high caliber and their role in underpinning SA-AKI research. From an authorship perspective, the research leadership within this discipline is predominantly confined to a select few remarkably prolific authors, notably Kellum J. A. from the United States and Bellomo R. from Australia. Not only do they lead in terms of publication volume, but they also demonstrate exceptional *h*-index performance, rendering them the most authoritative figures in this arena. Kellum J. A. investigates various dimensions of SA-AKI, encompassing concepts, epidemiology, pathophysiology, prevention, and treatment (7, 25, 29–32), and has generated a substantial corpus of seminal articles.

Co-cited literature refers to documents frequently referenced by multiple other publications; thus, it can be deemed a foundation of research within a field (19). In this bibliometric analysis, we selected the 10 most co-cited articles to illustrate the research landscape of SA-AKI. The most co-cited study, published by Singer et al. (1), revisited the definitions of sepsis and septic shock (33) established in 2001, reassessing and updating the pathobiology, therapy, and epidemiology of sepsis as necessary. These revised definitions and clinical standards afford greater consistency for epidemiological studies and clinical trials, facilitating the early identification and more timely management of septic patients or those at heightened risk of sepsis. Among these 10 co-cited papers, Hernando Gomez has authored two, with the initial review published in “Shock” in 2014. This review posits that AKI may manifest in numerous patients without overt indicators of systemic hypoperfusion, and that sepsis-induced AKI may occur even in the presence of normal or elevated renal blood flow. Consequently, renal injury may not be entirely elucidated by the classical paradigm of hypoperfusion, warranting consideration of alternative mechanisms. A “unifying theory” has therefore been proposed to elucidate the interplay between inflammation and oxidative stress, microvascular dysfunction, and the adaptive responses of tubular epithelial cells to septic injury, suggesting these responses are primarily adaptive and driven by mitochondria, ultimately elucidating the clinical phenotype of sepsis-induced AKI (7). The second review posits that while earlier perspectives suggested organ dysfunction ensues solely from hypoperfusion, this analysis contests that notion by asserting that AKI can arise alongside normal or augmented renal blood flow. Its characteristics are not confined to acute tubular necrosis or apoptosis, but instead manifest as heterogeneous, localized, gradual areas of peritubular capillary blood flow and oxidative stress within tubular epithelial cells. Furthermore, it proposes that microvascular dysfunction, inflammation, and metabolic responses to inflammatory injury might elucidate the fundamental pathophysiological mechanisms underlying acute kidney injury induced by sepsis (34). This realization is pivotal, as it opens avenues for a more profound understanding of the injury and repair processes, against the backdrop of decades of clinical trial outcomes. It also provides invaluable opportunities for the design of mechanism-targeted therapeutic interventions.

Peerapornratana et al. (25) published an article in “Kidney International,” redefining the conceptual framework, epidemiology, pathophysiology, prevention, and management of SA-AKI. They emphasized the current limitations in defining and diagnosing SA-AKI, acknowledging the potential of biomarkers as valuable supplements to clinical judgment, functional testing, and existing standards to enhance early detection, which may ultimately guide management and recovery monitoring. Nevertheless, effective and specific interventions for the prevention and treatment of SA-AKI remain sorely deficient. In summary, much-cited literature predominantly centers on elucidating the pathophysiology of SA-AKI, predicting novel biomarkers, and potentially refining treatment strategies to optimize outcomes for SA-AKI patients, yet these endeavors largely reside within the domain of fundamental research.

### 4.2 Research hotspots and frontiers

The references cited in the surge of citations concerning hotspots and frontiers represent burgeoning topics within specific research domains, as these references have been frequently acknowledged by scholars in recent years (35). To date, the majority of studies have indicated that SA-AKI is a grave condition wherein mitochondrial oxidative stress and inflammation play pivotal roles in its pathophysiology (36). Moreover, levels of cytokines (such as interleukin IL-6, IL-10, and macrophage migration inhibitory factor) demonstrate strong correlations with the onset of SA-AKI (37, 38), underscoring the significant influence of systemic inflammatory mediators in this process. Additional research suggests that sepsis-induced injury to renal tubular cells transpires through increased permeability resulting from endothelial disruption (39). Apoptosis is a form of programmed cell death, alongside necrosis, autophagy, and ferroptosis. Excessive apoptosis has been identified as a prominent characteristic of renal tubular cells in acute inflammatory environments, thereby facilitating the progression to chronic kidney disease through tubular atrophy and interstitial fibrosis (40). The trending topic map illustrates that the vanguard of acute kidney injury biomarkers is concentrated on mitochondrial dysfunction, inflammasome, ferroptosis, and macrophages.

#### 4.2.1 Mitochondrial dysfunction

Sepsis can instigate mitochondrial damage and immune dysfunction. Owing to perilous factors such as an abundance of reactive oxygen species (ROS) or nitric oxide within the inflammatory response, mitochondrial dysfunction, oxidative stress, and cellular apoptosis may be induced (41–43), ultimately culminating in cellular demise (44–46). Mitochondria, serving as the energy hub of organisms, partake in the oxidative metabolism of eukaryotes and represent the primary intracellular source of most reactive oxygen species (ROS) (47). ROS are regarded as pivotal agents inciting renal damage manifestations, including mesangial cell hypertrophy, podocyte apoptosis, glomerulosclerosis, and endothelial dysfunction. Furthermore, ROS function as significant mediators in proteinuria and compromise glomerular hemodynamics. Excessive ROS production linked to mitochondrial dysfunction may disrupt the equilibrium between ROS generation and the cellular defense mechanisms, thereby invoking oxidative stress (48), which is deemed a prerequisite for sepsis-associated acute kidney injury (SA-AKI) (49). Moreover, prior studies have indicated that mitochondrial dysfunction is not merely a vital factor inducing imbalanced oxidative stress within cells (50) but also one of the mechanisms by which sepsis inflicts damage upon various organs (51).

In sepsis, the generation of free radicals markedly escalates due to oxygen deprivation, incomplete oxidative reactions, and hypoxia. Mechanisms that hinder the antioxidant system further exacerbate mitochondrial dysfunction (52). Additionally, research suggests that morphological alterations in mitochondria serve as early indicators of ROS-induced mitochondrial dysfunction (53). Scholars increasingly acknowledge the role of mitochondria in the pathophysiology of acute kidney injury precipitated by sepsis and its potential as a therapeutic target (54).

Previous inquiries have elucidated that the Toll-like receptor 4 (TLR4)/nuclear factor-κB (NF-κB) signaling pathway constitutes a critical nexus mediating inflammation and is accountable for the initiation of mitochondrial dysfunction (55), recognized as one of the signaling pathways implicated in the onset of SA-AKI (56–58). Importantly, investigations have documented a profound association between TLR4/NF-κB activation and the occurrence of acute kidney injury, with the activation of this pathway likely paving the way for SA-AKI symptoms (59). It is posited that inhibiting the activity of the TLR4/NF-κB signaling pathway may attenuate oxidative stress and the synthesis of pro-inflammatory factors (60, 61). Current studies utilizing cecal ligation and puncture (CLP)-induced SA-AKI have demonstrated that TAK-242 can promote mitochondrial biogenesis (62), modulate mitochondrial quality (63), and ameliorate mitochondrial dysfunction (64), thereby inhibiting the TLR4/NF-κB signaling pathway, enhancing renal tissue mitochondrial function, and preventing CLP-induced SA-AKI in rats, providing substantive data support for the treatment of SA-AKI (65).

#### 4.2.2 Inflammasome

The inflammasome is a complex assembly of multiple proteins formed by cytoplasmic pattern recognition receptors (PRRs) and constitutes a pivotal element of the innate immune system. It identifies pathogen-associated molecular patterns (PAMPs) or danger-associated molecular patterns (DAMPs) derived from host sources, subsequently recruiting and activating the pro-inflammatory protease caspase-1. The activation of caspase-1 results in the cleavage of pro-IL-1β and pro-IL-18, culminating in the production of inflammatory cytokines such as IL-18 and IL-1β (66–68). Moreover, the activation of the inflammasome may instigate pyroptosis, a form of programmed cell death characterized by inflammation. Pyroptosis is a pro-inflammatory process of programmed cell death implicated in the pathogenesis of a variety of diseases, particularly kidney ailments. Focal cell death contributes to renal disorders through two primary pathways: the classical pyroptosis pathway mediated by caspase-1 and the non-classical pyroptosis pathway facilitated by caspase-11. The classical pyroptosis mediated by caspase-1 is a regulated mode of cell death that depends on the activation of caspase-1. Upon host infection, the generation of danger signals such as damage-associated molecular patterns (DAMPs) or pathogen-associated molecular patterns (PAMPs) occurs, which are detected by nod-like receptors (NLRs) that subsequently initiate pyroptosis (69, 70). NLRs commonly include NOD-like receptor family pyrin domain-containing protein 3 (NLRP3) or NOD-like receptor family pyrin domain-containing protein 4 (NLRP4). NLRP3, a cytosolic sensor, comprises a central NACHT domain, C-terminal leucine-rich repeats (LRRs), and an N-terminal pyrin domain (71), and is among the most crucial intracellular receptors that can be induced by NF-κB transcription. The activation of the NLRP3 inflammasome results in the maturation of caspase-1, facilitating pyroptosis and regulating the cleavage and maturation of pro-inflammatory cytokines like IL-1β and IL-18 (72), thus playing a vital role in the pyroptotic process (73–76). Research has demonstrated that the inhibition of NLRP3 inflammasome activation can mitigate the inflammatory response and the expression of kidney injury markers such as neutrophil gelatinase-associated lipocalin (NGAL) and kidney injury molecule-1 (KIM-1) in mice with lipopolysaccharide-induced acute kidney injury (77). Therefore, the NLRP3 inflammasome plays a significant role in the progression of acute kidney injury (78), albeit its precise role and mechanisms in sepsis-induced acute kidney injury remain poorly understood. Galectin-3, a member of the β-galactosidase family, plays significant roles in various biological processes such as cell proliferation, differentiation, adhesion, and apoptosis. The overexpression of Galectin-3 fosters renal cell apoptosis and the synthesis of type I collagen, thereby contributing to inflammation and fibrosis (79). The activation of NLRP3 induces intracellular oxidative stress, which in turn leads to an increased expression of Galectin-3 (80), suggesting that NLRP3 might trigger renal injury through galectin-3. Additionally, the production of reactive oxygen species (ROS) serves as a potential instigating factor for the assembly of the NLRP3 inflammasome (81). Due to factors such as infection, inflammation, or mitochondrial dysfunction, levels of mitochondrial reactive oxygen species (mtROS) rise. Oxidized mitochondrial DNA (oxmtDNA) is released into the cytoplasm, leading to the assembly and activation of the NLRP3 inflammasome via direct interaction with NLRP3 (82–84). Functioning as a sensor for mitochondrial dysfunction, the NLRP3 inflammasome highlights the connection between mitochondrial damage, autophagy/mitophagy, and inflammation (85). Studies have indicated that in a septic rat model, the caspase-1 inhibitor AC-YVAD-CMK significantly diminishes the expression of GSDMD in renal tissues. This inhibition of NLRP inflammasome expression reduces the pyroptotic death of renal tubular epithelial cells (RTECs), enhances antioxidant enzyme activity, decreases oxidative products, thereby providing protection against sepsis-induced acute kidney injury (86).

#### 4.2.3 Ferroptosis

Ferroptosis represents a novel variant of programmed cell death, first delineated in 2012 (87). This cellular demise is intricately governed by a myriad of metabolic pathways, encompassing redox homeostasis, iron metabolism, mitochondrial function, as well as the metabolism of amino acids, lipids, and glucose (88). In contrast to apoptosis, necrosis, and autophagy, ferroptosis is distinguished by the preservation of cellular membranes, nuclei that are both appropriately sized and dense, and diminutive mitochondria (89, 90). Ferritin heavy polypeptide 1 (FTH-1) holds a pivotal role in the maintenance of intracellular iron equilibrium and the modulation of ferroptosis (91). Moreover, it provides a protective function in cases of sepsis-induced organ failure (92). Ferroptosis serves as a pro-inflammatory mediator, attracting macrophages and inciting inflammation (93), which culminates in the augmentation of reactive oxygen species (ROS) and lipid peroxidation (94). As insights into ferroptosis expand, a plethora of studies has documented its activation during organ damage consequent to sepsis (95–97). Tubular ferroptosis is triggered in SA-AKI, corroborating recent findings (96, 98, 99). Furthermore, ferroptosis may accompany several processes, including ROS accumulation and lipid peroxidation, both of which contribute to acute kidney injury (AKI) (95, 100, 101). The formation of ROS is regarded as the paramount executor in ferroptosis, wherein an overabundance of ROS instigates oxidative stress, exacerbates mitochondrial dysfunction, and directly engenders renal injury (102). Elevated levels of ROS evoke inflammatory responses in distant organ damage (103). The kidney is particularly susceptible to ferroptosis. In prior studies, the genetic ablation of the ferroptosis regulator GPX4 resulted in acute kidney injury in murine models, ultimately culminating in mortality (104). Recent investigations have illuminated that, in AKI, the upregulation of the Hmox1 pathway performs an anti-ferroptotic function amid oxidative stress and inflammation (105). An inhibitor of mmu-miR-7212-5p enhances Hmox1 expression and mitigates ferroptosis by diminishing Acsl4 expression, suggesting that mmu-miR-7212-5p inhibitors may represent a promising clinical therapeutic target for sepsis-related AKI. The kidney is deemed one of the most susceptible organs during sepsis, in part due to the excessive rupture of erythrocytes induced by sepsis, which leads to the liberation of substantial quantities of free hemoglobin, heme, and iron into the bloodstream. The increased filtration and reabsorption of hemoglobin in the kidneys aggravate oxidative stress and ferroptosis (106). The infiltration of immune cells, particularly macrophages, also contributes to the accumulation of iron within tissues (107), resulting in oxidative stress and cellular injury (108). This aligns with previous reports indicating that FER1 alleviates LPS-induced organ injury by effectively counteracting membrane lipid damage through redox reactions (109, 110). Additionally, Glutathione peroxidase 4 (Gpx4) functions as a critical regulator of ferroptosis by inhibiting lipid peroxidation (111). Researchers have identified that AKI can result in the downregulation of endogenous H2S production, thereby diminishing glutathione (GSH) levels and amplifying cardiac oxidative stress (112). H2S has been extensively studied in various animal and cellular models of AKI, demonstrating efficacy in ameliorating renal damage (113). Sepsis may provoke ferroptosis through the elevation of mitochondrial lipid peroxidation and MDA levels, while concurrently reducing MMP and GSH levels (114). H2S may counteract AKI induced by iron toxicity by inhibiting mitochondrial oxidative stress. Iron-dependent cell death during SA-AKI exacerbates injury, indicating that the inhibition of ferroptosis could serve as a promising therapeutic strategy. The concept of iron death remains nascent within the realm of acute kidney injury research, and the interplay between iron death and other forms of programmed cell death will be a focal point for future investigations (115). Furthermore, iron death is also implicated in the transition from acute kidney injury to chronic kidney disease, and the regulation of iron death may prospectively avert this transition (116).

#### 4.2.4 Macrophage

M1 pro-inflammatory macrophages serve as the initial responders to septic kidney injury, possessing the capacity to clear damaged cells, promote renal fibrosis, and facilitate recovery (117). Dying tubular cells actively or passively release inflammatory mediators and damage-associated molecular patterns (DAMPs) into the extracellular microenvironment, thereby recruiting and fostering the differentiation of renal monocytes into M1 macrophages (118). The sustained presence of M1 macrophages can precipitate further tissue damage and worsen prognosis following kidney injury (119), while M2 macrophages play a pivotal role in suppressing inflammation and steering recovery (119). Extensive research emphasizes the significance of regulating M1/M2 macrophage polarization as a promising therapeutic target for acute kidney injury (AKI) (120, 121). Moreover, macrophages have been identified as critical immune cells in the process of ferroptosis (122). Prior reports suggest that iron-dependent cell death in tumor cells promotes M1 macrophage polarization and contributes to pro-inflammatory responses (123, 124). Hence, it may be inferred that macrophages, particularly M1 macrophages, might be involved in exacerbating apoptosis-related inflammation during kidney injury. C-type lectin domain family 4, member E (Clec4e, also known as Mincle), is markedly expressed on the surface of M1 macrophages, detecting pathogens and endogenous ligands, thereby initiating innate immunity in the realms of host defense, immune disorders, infectious diseases, inflammation, and even tumors (125). Studies have indicated that in ischemia/reperfusion-induced AKI, Mincle and its downstream mediator, the Syk pathway, are integral in maintaining the M1 polarization of macrophages, promoting inflammation, and aggravating renal damage (126–128). Inhibiting Mincle expression in macrophages has been demonstrated to significantly alleviate inflammation, suggesting a potential target for AKI treatment (129). Splicing factor 130 (SAP130), a component of the small nuclear ribonucleoprotein, is released from dying cells during processes such as apoptosis and regulated necrosis (130, 131). It is identified as the first described endogenous ligand of Mincle, inducing the activation of the M1 phenotype in macrophages during tissue injury (128, 130, 132). Prior studies have shown that M1 macrophages recognize SAP130, activating Mincle and contributing to various ailments, including acute and chronic kidney injuries (133, 134). SAP130 released from iron-dead tubular epithelial cells (TECs) further activates the Mincle/Syk/NF-κB signaling pathway in macrophages, propelling the formation of M1 macrophages, ultimately exacerbating TEC iron death and initiating an inflammatory feedback loop within the microenvironment of sepsis-associated acute kidney injury (SA-AKI). Silencing Mincle in macrophages or neutralizing SAP130 disrupts the crosstalk between TECs and macrophages, both *in vivo* and *in vitro*, thereby reducing tubular cell death and interstitial macrophage infiltration. This intervention mitigates renal injury and preserves renal function. Numerous studies highlight that the interplay between tubular epithelial cell (TEC) death and macrophage-mediated inflammation is crucial in the progression and reparative mechanisms of AKI (135, 136). However, macrophage phenotypes and functions exhibit considerable plasticity, potentially exerting opposing roles in both AKI and repair processes by altering their phenotypic expression (137). Consequently, an enhanced understanding of macrophage activation following tubular injury is essential.

## 5 Advantages and limitations

This study employed bibliometric methods to conduct the first comprehensive visualization analysis of research on SA-AKI, thereby assisting scholars in better comprehending the field’s focal points and trends. Furthermore, we concurrently employed three bibliometric tools for our investigation, ensuring objectivity in our data analysis process. Ultimately, bibliometric analysis yields more profound insights into hotspots and frontiers compared to traditional reviews. Nonetheless, our study is not without limitations: firstly, we exclusively analyzed English articles indexed in the Web of Science Core Collection within a defined timeframe, which may have led to the omission of pertinent research from other databases. Secondly, bibliometric analysis tools may possess intrinsic limitations and biases that could potentially influence the results. Nevertheless, the use of visual methods to discern the current status, hotspots, and trends within a field remains invaluable.

## 6 Conclusion

In summary, this bibliometric analysis meticulously examines the present status of research on SA-AKI, charting the trajectory of future developments in the field. The swift rise in publications signifies an escalating global interest among scholars in SA-AKI. Prominent nations in this research endeavor include China and the United States; nonetheless, there exists a pressing need to enhance collaboration and communication among countries and institutions. Investigations centering on mitochondrial dysfunction, iron metabolism pathways, and the identification of antagonistic axes in the pathogenesis of SA-AKI are emerging as pivotal areas for forthcoming studies. Therefore, delving into the pathophysiology and therapeutic strategies of SA-AKI offers considerable promise for precise treatments in the future. This study may assist scholars in obtaining a clearer and more rapid comprehension of the global research landscape surrounding SA-AKI, thereby providing valuable insights for institutions or groups pursuing research collaborations.

## Data Availability

The original contributions presented in the study are included in the article/supplementary material, further inquiries can be directed to the corresponding author.

## Footnotes

^1^ https://www.bibliometrix.org
